# Exploring the impact of virtual leadership on job satisfaction in the post-COVID-19 era: The mediating role of work–life balance and trust in leaders

**DOI:** 10.3389/fpsyg.2023.994539

**Published:** 2023-03-14

**Authors:** Hala Koleilat Al Dilby, Panteha Farmanesh

**Affiliations:** Department of Business Management, Faculty of Business and Economics, Girne American University, Girne, Türkiye

**Keywords:** post COVID-19, psychological behavior, virtual leaders, employee wellbeing, positive work outcome, work–life balance, trust in leader

## Abstract

Leadership remains a highly important role in the management of employees' psychological and physical well-being, particularly in the aftermath of the COVID-19 pandemic. As various sectors adapted virtual settings to overcome the restrictions posed by the pandemic, the vitality of virtual leaders became more pivotal as they could enhance the virtual work environment for employees and steer teams toward organizational goals. This study assessed the effect of virtual leaders on employees' job satisfaction in the information technology sector as a high-performance industry. Furthermore, the mediating effects of trust in leaders and work–life balance on the virtual leadership–job satisfaction relationship were assessed in the proposed model of this research. Through a deductive quantitative approach and using purposive and convenience sampling techniques, a total of 196 respondents participated in the research. The data analysis process was deployed *via* Smart PLS software and the PLS-SEM technique. The results showed that virtual leaders play a major role in determining information technology (IT) employees' job satisfaction while the mediating effects of both trust in leaders and work–life balance are significant factors that can enhance the work environment for leaders to achieve better results. The statistically significant findings of this research suggest a number of positive work outcomes and pathways with scholarly and managerial implications that can be beneficial for leaders in relevant sectors.

## Introduction

The current research aimed to examine the relationship between virtual leadership and job satisfaction as a topic of interest, especially during and after the occurrence of the COVID-19 global pandemic. The conduct of this study follows the recent string of studies in the literature that address leadership and its influence in the aftermath of a global crisis (e.g., Bouwmeester and Kok, 2018; Qiu and Dauth, 2021; Zacher and Rudolph, 2021). As numerous industries were affected by the outbreak of the pandemic, the role of leaders in maintaining a healthy, functional, and caring environment for employees became more vivid. This research primarily focused on the impact of virtual leaders and their capabilities and skillsets that appropriately match online settings now commonly used by businesses across the world. In addition, the current research aimed to analyze the mediating effects of work–life balance and trust in leaders as influential elements that can improve the work outcomes desired by leaders (e.g., job satisfaction) (Krug et al., 2021; Rashmi and Kataria, 2021; Singh, 2021). In this sense, this research focused on the role of leaders using modern methods and approaches to impact human resource management (HRM) initiatives in times of crises through virtual work tools and environments (Collings et al., 2021). Among the impacts of significant crises, such as the global pandemic (with its vast effects on all aspects of businesses), stress, anxiety, uncertainty, decreased engagement, changes in work, and lowered job satisfaction have been reported by recent studies across various fields (Bailey and Breslin, 2021; Castellano et al., 2021; Collings et al., 2021; Rashmi and Kataria, 2021; Rigotti et al., 2021; Rudolph et al., 2021). This research demonstrated that virtual leaders are capable of increasing the job satisfaction of information technology (IT) employees by establishing trust and fostering work–life balance, especially in the current status of the business world where leadership skills in virtual realms are greatly needed. This in turn can result in positive work outcomes through enhanced psychological behavior of individuals within a firm. This research argues that the aforementioned work outcomes can be greatly influenced by virtual leaders, particularly in the post-COVID-19 era. Both scholars and managers can benefit from such studies through the theoretical contributions and managerial implications that can be used to enhance employees' experience within virtual workplaces.

The literature shows a gap regarding leadership and its linkage to job satisfaction in the virtual era where more businesses are currently functioning due to the shifts caused by the COVID-19 pandemic. Therefore, the current research aimed to address a number of gaps in the extant literature by building on recent findings regarding positive work outcomes and organizational psychology. In doing so, this research contributes to leadership, HRM, organizational behavior, and organizational psychology in terms of theory (e.g., Bouwmeester and Kok, 2018; Koziel et al., 2021; Qiu and Dauth, 2021; Rashmi and Kataria, 2021; Zacher and Rudolph, 2021; Farmanesh and Zargar, 2023). Accordingly, this research aimed to contribute to the practical domain for decision-makers (i.e., leaders) to better understand the influential elements for enhancing the job satisfaction of their employees. As a result, firms maintain their competitive advantages during and in the aftermath of the COVID-19 pandemic. Virtual leaders can thrive in sectors that are reliant on virtual settings and are highly demanding such as the IT sector. Employees in this sector are required to have a number of skills that are technical, methodical, and analytical, which, combined with high workloads, can negatively affect employees' wellbeing (Qiu and Dauth, 2021; Farmanesh and Zargar, 2023). We observed that virtual leaders can tap into their characteristics and enhance the virtual workplace for the satisfaction of employees in the high-performing IT industry so that they are empowered to accomplish a large number of projects. In doing so, trust, work–life balance, resilience, competence and skills, assistive programs, and counseling services are important matters that this research looked into from the perspective of IT consultants and employees' wellbeing.

Some sectors such as IT, (digital) marketing, data science and related businesses, legal advising, accounting, banking, management, and strategic consulting are high-performance demanding jobs that can be project-based with a large number of associated tasks (Mühlhaus and Bouwmeester, 2016; Alvesson and Einola, 2018). As the requirements of such jobs are specific and require high performance, it is important to note that, when the pandemic started, these businesses faced an increase in demand for services and products from customers (Bouwmeester and Kok, 2018; Qiu and Dauth, 2021). Notably, many individuals with IT-related jobs can have several projects with varying deadlines that can negatively impact work–life balance. This can be linked to context variation from one project to another (Pinto et al., 2014), requiring the employees to adjust to different work settings due to distance and/or time difference (Mühlhaus and Bouwmeester, 2016). Burnout, turnover intentions, stress and anxiety, and other negative outcomes of virtual work intensity can pose threats to individuals' wellbeing, especially in times of crises (i.e., COVID-19 pandemic) (e.g., Cho and Park, 2021). These can be further connected to decreased social interactions and other stress-related aspects of their work situations that hinder job satisfaction in virtual work environments (Bulińska-Stangrecka and Bagieńska, 2021).

Within the context of current research, the literature shows that there is a tendency to develop and/or hire leaders who demonstrate resilience in terms of project continuation and distance/virtual leadership. Furthermore, the ability to use various tools to communicate and influence the behavior of employees is a key element, which has become more vivid during the crisis of the COVID-19 pandemic (Kniffin et al., 2021; Rudolph et al., 2021) and has remained essential in its aftermath, as many sectors are now functional with virtual work elements. In this respect, leaders' influence is a significant factor due to its impact on desirable/positive work outcomes within the context of organizational psychology (i.e., trust and satisfaction). Therefore, leaders who focus on wellbeing, values, behaviors, and attitudes of their followers are more desirable in the current state of many businesses. Virtual leaders are under examination in this study as their characteristics fit the status quo during the COVID-19 health crisis, and such traits can be applied after the pandemic. By focusing on aspects such as trust, individualized care, and attention, as well as consideration for work–life balance and overall employee wellbeing, these leaders can positively influence job satisfaction. This is vital as a psychological factor for individuals and as a predictor of a number of positive organizational behaviors and work outcomes (Klebe et al., 2021; Terkamo-Moisio et al., 2021; Zacher and Rudolph, 2021; Farmanesh and Zargar, 2023).

In addition to the aforementioned gaps in leadership research after the pandemic, IT employees' job satisfaction, the region of the Middle East, and empirical evidence covering this subject, this research builds on the work of Qiu and Dauth (2021) by following their recommendations of using structural equation modeling (SEM) as it uses the PLS-SEM to analyze the mediating effect. Selecting a measurement scale for job satisfaction that does not overlap with other constructs further contributes to the literature on organizational behavior and the core concept of HRM as a domain of science and management. Similarly, Terkamo-Moisio et al. (2021) reported that trust, virtual communication, and positive team culture are some of the main characteristics of virtual leaders. Given the telecommuting phenomena and the leaders' roles within such, this research uses the term virtual or E-leadership to refer to leaders who function in virtual workplaces. Transformational leadership can foster trust, well-established collaboration, high performance, wellbeing, and a shared vision with a clear path toward goals (Li et al., 2016; Maduka et al., 2018; Ramserran and Haddud, 2018; Farmanesh and Zargar, 2023). Such leaders can enhance job autonomy, provide support (e.g., training, counseling, and personal development practices), constantly seek new ideas related to digital development, and demonstrate the ability to learn. These characteristics appear to be linked to their understanding, awareness, technological savviness, and problem-solving ability in complex organizational structures (Poulsen and Ipsen, 2017; Terkamo-Moisio et al., 2021). In turn, positive work outcomes and a positive work environment can be achieved within the firm, which benefits employees and the firm alike. In light of the noted gaps and directions, the current study aimed to provide tangible empirical evidence that can be beneficial for scholars as well as leaders in high-performing industries to better steer their firms in future crises after the COVID-19 pandemic.

Based on the aforementioned aims and context, the research questions were as follows: (a) Can virtual leaders improve the job satisfaction of IT employees?; (b) Does trust in the leader mediate the relationship between virtual leaders and employees' job satisfaction?; and (c) Is there a mediating effect from work–life balance on the virtual leadership-job satisfaction linkage? The following sections include the theoretical framework of the research and hypotheses development, where the foundation of the study is described. The study is then followed by sections describing the research design, sampling procedure, and measurements. In this stage, data analyses were conducted and the results are presented. The study is then finalized with discussions of the findings, conclusions, and limitations/recommendations.

## Theoretical development

### Virtual leaders and job satisfaction

Virtual workspaces are a key tool for modern businesses, especially in industries such as education, business and marketing, IT, and other high-performing sectors (Bouwmeester and Kok, 2018). They enable firms to adjust to the market in terms of speed, adapting to changes, and reducing costs (Qiu and Dauth, 2021). The concept of virtual work has been examined and, in relation to job satisfaction, the literature demonstrates a lack of consensus across different industries (Mansfield, 2018; Schall, 2019). A number of factors are essential for the determination of job satisfaction as a psychological construct (e.g., coworkers, leaders, compensation, stress, support, and work intensity) (Hong et al., 2016; Cho and Park, 2021; Telyani et al., 2021). Virtual work is explained as a work setting that does not rely on a fixed time or place and uses Information and Communication Technology (ICT) for interaction (Qiu and Dauth, 2021). Telecommuting (being distant from the central office for work), virtual teams (location or time-distant dynamics), and computer-mediated work (CMW) (problem-solving, decision-making, and productivity) have been reported as the main streams of research in this context (Raghuram et al., 2019).

**Figure 1:**
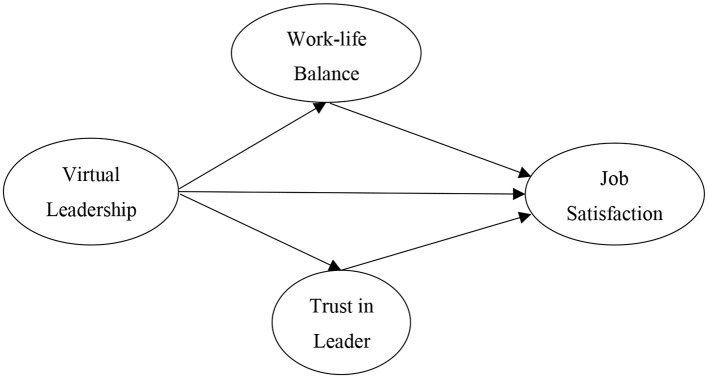
Theoretical model.

Job satisfaction can be described as the direct relationship between an individual's expectations of their job and actual experiences (Locke, 1976). Within the context of virtual work environments, the Social Presence Theory (Short et al., 1976) explains the low level of in-person interactions and high competence in technology, which is further explained by the Media Richness Theory (Daft and Lengel, 1986). Crucial to the focus of this research, virtual work has been noted to lack certain sources for establishing trust, career development practices, and thus meet individuals' expectation levels in a satisfactory way (Bulińska-Stangrecka and Bagieńska, 2021; Qiu and Dauth, 2021; Rashmi and Kataria, 2021). The current research aimed to investigate the role of virtual leaders and their characteristics in fostering an atmosphere of trust, where job satisfaction can be positively affected. In addition, within the context of virtual work environments, this research included the mediating effect of work–life balance in the proposed model (Figure 1).

In accordance with what has been mentioned regarding the context of leadership and employees' work outcomes, Maslow's Hierarchy of Needs can be embedded in relation to virtual teams and the role of leaders in affecting job satisfaction. The need for improvement and individuals' tendency toward progress is emphasized by leaders to improve the experiences of employees and thus, increase job satisfaction. We assumed that effective virtual leaders possess the characteristics that, through self-actualization theory, can guide employees toward personal and professional development (Maslow, 1981). In this sense, leaders can tap into the power of HRM and provide support systems and professional development courses that structure career paths and, as a result, increase job satisfaction (Barnett, 2017; Bhatnagar and Grosse, 2019). Due to the vast impacts of the COVID-19 crisis, various threats have been posed to the health and wellbeing of people across the world (Zacher and Rudolph, 2021), making the role of virtual leaders more important. Accordingly, disciplines such as HRM, organizational behavior, organizational psychology, and industrial aspects have focused on this subject (e.g., Rasool et al., 2019; Kniffin et al., 2021; Meyer et al., 2021; Rudolph et al., 2021; Syrek et al., 2021; Zacher and Rudolph, 2021).

As work environments have changed (even for IT companies as offices were closed and team settings were no longer used or available), communication has been negatively impacted. As previously noted, job satisfaction and work–life balance can significantly change with regard to virtual work situations and its management/leadership (Dhamija et al., 2021; Qiu and Dauth, 2021). While resilience and flexibility are positive outcomes of this shift, there are various psychological elements that a virtual leader should take into account. Positive influence on others' behaviors, attitudes, and feelings on an individual basis are key factors (Sedrine et al., 2020; Wang et al., 2022). Notably, E-leaders have to cope with virtual work-specific challenges as well as those of traditional team leadership (Farmanesh and Zargar, 2023). Due to the lack of physical interactions, virtual leaders should further tap into different positive and ethical leadership styles (i.e., transformational and servant) through different approaches (Multifactor Leadership) (Avolio et al., 2000; et al., 2014; Liao, 2017). In this sense, as employees experience higher rates of danger from the pandemic, stress, anxiety, and conflict may arise, which further require E-leaders to be capable of managing such environments.

Transformational and servant leadership precede virtual leadership, which implies that such leaders are constantly seeking positive outcomes while fostering the wellbeing of individuals. Thus, coordination and establishment of trust and a trustworthy work environment, conflict management, shared vision and goals, and the ability to solve complex organizational issues are the key characteristics of E-leaders (Singh, 2021; Farmanesh and Zargar, 2023). Theoretically, demands (strain) and resources (motivation) are the two dimensions of the Job Demand-Resources Model (JD-R) (Demerouti et al., 2001). The JD-R model fits in the scope of current research as it addresses the high demands of the IT sector for employees in terms of workplace demands (e.g., skill, time, accuracy, and meeting deadlines). Furthermore, the JD-R model encompasses the intangible elements of the job (e.g., work–life balance, loneliness, stress, and satisfaction) that can have low resources as employees might lack the equipment or adequate space for conducting their jobs (particularly after the pandemic as individuals' wellbeing needs to be cared for). To motivate employees in virtual settings, a leader is to appropriately address both the demands and resources of the job to improve the environment for their employees. As the pandemic had severe impacts on work characteristics such as a reduction of interaction and cognitive resources, increased stress, job insecurity, and work–life balance (Zacher and Rudolph, 2021), the JD-R model can be used to explain the work-related elements and the employees' behavioral and/or work outcomes. Various studies have reported social support, job autonomy, organizational support, and HRM practices for wellbeing and similar initiatives to positively influence employees' satisfaction in times of crises (Bhatnagar and Grosse, 2019; Castellano et al., 2021; Meyer et al., 2021). This study follows a relatively new string of literature that addresses virtual leaders, workspaces, and the behavioral factors of virtual teams. Accordingly, a hypothesis was shaped as follows:

- *Hypothesis 1: Virtual leadership is positively related to the job satisfaction of IT workers*.

### Trust in leader as a mediator

The crisis management and resilience framework (Williams et al., 2017) provides an explanation of the anticipation and responses of societal groups (individual, organizational, and communities) in the face of an extreme event (i.e., the COVID-19 pandemic). Thus, resilience is the ability to adjust and keep a satisfactory level of functionality during and upon the occurrence of a crisis. The current research further used the Social Identity Theory and its subsequent identity change model to address the vast changes the COVID-19 pandemic has caused in the wellbeing of individuals (Haslam et al., 2021; Zacher and Rudolph, 2021). Death, illness, job loss, sudden changes in work, and lack of social interactions (psychosocial relationships) are among the aspects of life that have been diminished by the pandemic and are addressed through the social identity continuity and gain pathways (see Haslam et al., 2021). Similar studies have referred to this theory with regard to employees' perceptions during the COVID-19 pandemic (e.g., Krug et al., 2021; Qiu and Dauth, 2021; Singh, 2021). This study focused on the importance of trust in creating an atmosphere for employees in which they can have their lives positively impacted by the actions their leaders undertake.

Virtual leaders are aware of numerous aspects of managing remote workforces. Therefore, by performing actions to positively manage a sense of identity, motivation and needs, proper communication, and the lack of social interactions, a leader can establish an adequate workspace. In this sense, trust-building is a consequence of leaders' strategies to achieve organizational outcomes. It is important to note that trust in virtual settings may be temporary (Farmanesh and Zargar, 2023). Time, culture, distance (location), and manner of interactions are key elements in trust creation among virtual team members, which is the key aspect of a leader's role (Zargar et al., 2019; Sedrine et al., 2020). Linked to transformational leadership (Bass and Avolio, 1993) and servant leadership (Greenleaf, 1998), virtual leaders' effectiveness could be measured by establishing collective trust. Developing and maintaining trust relationships can be achieved by creating personal bonds with individuals, showing concern and care for the needs of members, exhibiting respect and fairness, fulfilling promises, and providing professional assistance in times of uncertainty and need are the characteristics of an ethical and positive leadership approach for job satisfaction. High levels of trust imply that staff will share issues with their leader which provides a certain level of job satisfaction and leads to its optimization by the leader (Zargar et al., 2019). As a vital psychological factor, trust development should be the focus of leaders within organizations (Rezvani et al., 2016) as it is a solid element for the linkage between individuals and their organizations (Meng and Berger, 2019). The existence of trust can lead to success for the firm through sustainable competitive advantages among its staff (Yuan et al., 2018). Accordingly, the current research demonstrated that employees' trust in the leader has a mediating effect on job satisfaction, which is shown by the following hypothesis:

- *Hypothesis 2: Trust in leaders mediates the linkage between virtual leaders and the job satisfaction of IT employees*.

### Mediating role of work–life balance

Work–life balance (WLB) addresses the existence of a conflict between one's role at work and the role outside work (Frone et al., 2003; Bouwmeester and Kok, 2018). While there are various definitions for the concept of WLB, in the context of this research, it was regarded as the direct conflict between the work domain and the personal domain (Frone et al., 2003). WLB encompasses all domains of life and their interaction with the sphere of work. Job satisfaction can be significantly impacted by this factor as high demands or conflicts in life or between life and work can cause stress, burnout, turnover intentions, lack of engagement, and a decrease in job satisfaction (Lewis et al., 2007; Mazmanian et al., 2013). Individual and organizational elements can cause variations in WLB (e.g., high-performing demands, project-based work, work overload, deadline conflicts, family issues, and having to take care of parents or family members). Referring to the work of Bouwmeester and Kok (2018), clients can also affect WLB as their demands or compensations vary and require adjustments to the specific needs of several clients at the same time. In this regard, employees in the IT sector face a high number of clients and often work on a project basis (as part-time employees), which can reduce their job satisfaction (Singh, 2021). According to a number of findings, IT employees are occupied within a highly competitive market that requires high performance, high demands, quality services, and longer than average weekly working hours (e.g., Lupu and Empson, 2015; Mühlhaus and Bouwmeester, 2016; Blagoev et al., 2018).

As new technologies cross the boundaries of time and place, WLB can be diminished due to connectivity at all times (Currie and Eveline, 2011) that blurs the lines between family and work hours. This can be further linked to the job demand-resource model (JD-R), which encompasses the high demands of jobs in the IT sector as well as the limitations of resources available to the employees. Organizational support through leaders and the establishment of a positive work atmosphere, where WLB is highly considered and thus the wellbeing of individuals is cared for, can lead to higher job satisfaction as WLB can act as a mediating variable (Bouwmeester and Kok, 2018; Rashmi and Kataria, 2021). Leaders can provide various means for their followers to enhance their WLB and thus, impact their job satisfaction. Specific means to enhance employees' WLB may include WLB support policies and arrangements, negotiable holidays or different work settings, telecommuting, flexibility in working time, fitness and healthcare programs, and professional development practices (Smith, 2010; Mayerhofer et al., 2011; de Janasz et al., 2013). Leaders and peers can fill the gap of social support during times of crises such as the COVID-19 pandemic, thereby affecting WLB in a positive manner by developing a trust culture among team members or leaders (team-member exchange and leader–member exchange theories) (Seers, 1989; Liden et al., 1997).

In addition to the previously mentioned means to facilitate WLB, compensation, non-monetary bonuses, and other incentives can positively impact the perception of employees regarding WLB, where non-monetary incentives have been reported to be of higher significance for increasing job satisfaction (Nelson and Todd, 2004). Following the recommendations of Bouwmeester and Kok (2018), this study takes a quantitative approach to define the linkage among the constructs of the hypothesized model (Figure 1). As the core concept of WLB is to reduce conflict between the two realms, it has vivid impacts on wellbeing and job satisfaction in a variety of different cultures, stating its generalizability (Haar et al., 2014, 2017; Braun and Peus, 2018). Virtual leaders (through their aforementioned characteristics) can build relationships based on trust by genuinely considering the ideas and thoughts of their followers, following ethics (e.g., fairness), focusing on wellbeing and WLB, and providing the necessary means to fill the gap of direct interactions. As a result, job satisfaction tends to increase among their followers. This is based on the predictors of WLB linked to leadership behavior and characteristics (e.g., Haar et al., 2017; Bouwmeester and Kok, 2018). Based on this argument, the current research posed the following hypothesis:

- *Hypothesis 3: Work–life balance can mediate the linkage between virtual leaders and IT employees' job satisfaction*.

## Research methodology and approach

The hypotheses of this research are illustrated in Figure 1. In this sense, the sampling procedure and measurements used in this research were selected for the specifics of the current model. This model developed the theoretical application of virtual leadership models while providing empirical evidence regarding virtual work behavior in the era after the COVID-19 pandemic. The current study used a quantitative and deductive approach through a survey that was derived from available and valid scales in the extant literature and administered by the researchers. This approach enabled the researchers to measure employees' job satisfaction with regard to the behavior of leaders within a given organization. As employees were the sample data of this research, the deployed approach allowed for the measuring and understanding of perceptions and understanding of employees regarding virtual leadership role and its impact on their job satisfaction. Furthermore, due to the purposiveness of sampling criteria, the respondents could be considered adequate in terms of representativeness. Lastly, through the established professional networks of the researchers, the accessibility required for collecting such samples was made available.

### Sampling procedure

The data for this research were collected from the IT personnel of several companies that, due to the COVID-19 pandemic, have completely shifted to virtual workspaces. These firms varied across various industries (e.g., finance, real estate, trade, data analysis/data companies). Following the recommendations of Hair et al. (2017), a sample size of over 183 was calculated as appropriate for this study (statistical power = 80%, Min R2 = 0.10, and α = 0.01). Before the main data collection, a pilot study was conducted with 20 participants to ensure the readability and validity of all the items included. Managers of various companies were contacted through emails and phone calls to establish the ethical and disciplinary means for data collection. A criterion was used to ensure that virtual leadership was embedded within the selected firm and that each firm had official WLB policies for employees that were established during and after the pandemic. A total of 250 questionnaires were distributed through email (using a convenience and purposive method based on the inclusion criteria), of which 196 were received for final data analysis after data screening. A total of 35 questionnaires were returned and 19 responses were incomplete (the missing data with more than 10% missed responses were removed from the final dataset), resulting in a 78.3% response rate with < 60% of the questionnaires containing valid information. The participants were from all project-related departments and various seniority levels. Data confidentiality disclosure and exclusion of sensitive information (e.g., income, name, and religion) were noted to reduce the common method bias (Podsakoff et al., 2003; Asghar et al., 2022; Rasool et al., 2022). Table 1 provides a report on the respondents' profiles.

**Table 1:** Respondent profile.

**Demographics**	**Categories**	**Frequency**	**(%)**
Age	20–25	78	39.79
	26–31	65	33.16
	32–37	32	16.33
	38 and older	21	10.72
Gender	Men	97	49.49
	Women	99	50.51
Education	Bachelor	45	22.96
	Masters	118	60.20
	PhD	33	16.84
Marital Status	Single	109	55.61
	Married	87	44.39

### Measurement

The virtual leadership scale from the work of Avolio and Bass (2004) in accordance with the extant literature was used to thoroughly measure leader behavior and effectiveness. A short version of the Multifactor Leadership Questionnaire (MLQ) is used, with a sample item being: “*The leader treats me as an individual rather than just a member of a group”*. On the same note, trust in the leader was adapted from the Organizational Trust Inventory (OTI) developed by Cummings and Bromiley (1996), with a sample item being: “*The manager seeks to gain trust from everyone”*. Furthermore, the job satisfaction scale was derived from Macdonald and Maclntyre (1997); a sample item is: “*I feel good about working in this organization”*. The work–life balance scale was taken from the work of Haar (2013); items included, for instance, “*I manage to balance the demands of my work and personal life equally”*. All items were designed on a 5-point Likert scale using strongly agree = 1, agree = 2, neutral = 3, disagree = 4, and strongly disagree = 5. Gender, age, education level (Nelson, 2008; Qiu and Dauth, 2021), and marital status (Saner and Eyüpoglu, 2013) were considered the control variables to control for unwanted effects on job satisfaction.

### Analysis

Descriptive statistics regarding the respondents' profiles are presented in Table 1.

Table 2 represents the means, standard deviation, and correlation among all measurements. Table 3 shows the results of applying the measurement model on the cross-sectional data of this research, which showed a satisfactory level of outer loading (>0.71), internal consistency (alpha and Rho A; >0.7), composite reliability (CR), and average variance extracted (AVE; >0.5), indicating the validity and consistency of the selected measures (Chin, 2010; Hair et al., 2016) and discriminant validity (Henseler et al., 2015) (heterotrait-monotrait (HTMT) >0.85) values, which are provided in Table 4.

**Table 2:** Means, SD, and correlation of measures.

**Latent variable**	**Mean**	**SD**	**VL**	**WLB**	**TIL**	**JS**
VL	3.6541	1.03264	1			
WLB	3.4580	1.12310	0.587^**^	1		
TIL	3.3695	1.23148	0.674^**^	0.321^**^	1	
JS	3.3154	1.10943	0.691^**^	0.593^**^	0.588^**^	1

n = 196, VL, virtual leadership; WLB, work–life balance; TIL, trust in leader; JS, job satisfaction.

^**^Correlation is significant at the 0.01 level (two-tailed).

**Table 3:** Measurement model.

**Constructs**	**Indicators**	**Outer Loadings**	**Alpha**	**Rho A**	**CR**	**AVE**
VL	VL1	0.711	0.817	0.812	0.811	0.610
	VL2	0.842				
	VL3	0.950				
	VL4	0.743				
	VL5	0.754				
WLB	WLB1	0.801	0.860	0.868	0.840	0.722
	WLB2	0.823				
	WLB3	0.819				
	WLB4	0.834				
	WLB5	0.830				
TIL	TIL1	0.841	0.883	0.872	0.873	0.581
	TIL2	0.855				
	TIL3	0.888				
	TIL4	0.778				
	TIL5	0.897				
JS	JS1	0.846	0.801	0.823	0.810	0.712
	JS2	0.863				
	JS3	0.708				
	JS4	0.721				
	JS5	0.876				

**Table 4:** Heterotrait–monotrait (HTMT).

	**VL**	**WLB**	**TIL**
VL			
WLB	0.624		
TIL	0.739	0.445	
JS	0.598	0.480	0.533

Associated paths in the theoretical model were analyzed in terms of a structural model using the bootstrapping technique (1,000 resamplings) to measure the model accuracy of PLS, which are shown in Table 5 alongside hypotheses testing.

**Table 5:** Structural model and hypothesis testing.

**Effects**	**Relations**	**β**	**t-statistics**	**F̧^2^**	**Decision**
**Direct**					
H1	VL → JS	0.302	5.201^***^	0.115	Supported
**Mediation**					
H2	VL → TIL → JS	0.229	2.740^**^	0.032	Supported
H3	VL → WLB → JS	0.207	2.571^*^	0.026	Supported
**Control Variables**					
	Gender → JS	0.131	2.465^*^		
	Age → JS	0.102	2.059^*^		
	Education → JS	0.121	2.124^*^		
	Marital status	0.125	2.044^*^		

${\text{R}}_{\text{TIL}}^{2}$ = 0.32 / ${\text{Q}}_{\text{TIL}}^{2}$ = 0.24.

${\text{R}}_{\text{WLB}}^{2}$ = 0.31 / ${\text{Q}}_{\text{WLB}}^{2}$ = 0.19.

${\text{R}}_{\text{JS}}^{2}$ = 0.52 / ${\text{Q}}_{\text{JS}}^{2}$ = 0.41.

SRMR: 0.024; NFI: 0.925.

^*^0.05, ^**^0.01, ^***^0.001.

## Discussions

The results presented in the tables support the hypotheses of the research (see Table 3). The R-square values show the variations in job satisfaction explained by the independent variables, and the Q-square values show the predictive relevance of the model fit, which further implied a satisfactory level for the structural model. This supported the first hypothesis (β = 0.302, *t* = 5.201), which indicated a significant linkage between virtual leaders and job satisfaction. While similar reports have been noted in the extant literature (e.g., Bulińska-Stangrecka and Bagieńska, 2021; Qiu and Dauth, 2021; Rashmi and Kataria, 2021), the current results highlighted the importance of virtual leaders in the high-demanding IT sector. This was followed by the acceptance of hypothesis 2 (β = 0.229, *t* = 2.740), which exhibited the mediating effect of trust in a leader, implying its significant influence on the outcomes of the workplace (i.e., job satisfaction). This factor has been found to be highly influential in improving job satisfaction among employees in various sectors that can be categorized as high-performing industries, such as tourism, IT, and education (Zargar et al., 2019; Haslam et al., 2021; Zacher and Rudolph, 2021; Zaman et al., 2022).

Effective virtual leaders can obtain desirable outcomes through trust-building with their teams although it can be harder to obtain *via* virtual settings. Focus and emphasis are required from the leader to establish an environment of trust. Similarly, hypothesis 3 was supported (β = 0.207, *t* = 2.571). This suggested that WLB had a mediating effect on the relationship between virtual leaders and the job satisfaction of their employees. These findings demonstrated that employees could have a higher satisfaction level toward their demanding, specialized, and high-performing jobs through adequate leadership approaches (i.e., virtual) that take the wellbeing of their employees into consideration. This leads to the establishment of work settings that enable autonomy and the creation of balance among the domains of life for individuals. Notably, the *p*-values of all the hypotheses were below 0.05, implying their statistical significance. In addition, the NFI and SRMR values showed a model fit due to their value (Henseler et al., 2015).

## Conclusion

The results of this study agreed with prior research in relevant fields while contributing to the current understanding of the role of virtual leaders in the high-performance sector of IT since the COVID-19 pandemic. In this sense, the current findings indicated the mediating effect of WLB during times of crisis on the job satisfaction of IT employees. This was linked and embedded within the pivotal role of virtual leaders in incorporating WLB-specific initiatives for the wellbeing of staff, particularly in accordance with their personal needs after the COVID-19 pandemic (Rashmi and Kataria, 2021). Moreover, the social identity model of identity change was developed in this research, as important factors such as vast changes at work and death or illnesses of relatives included in the WLB programs of the company implied by virtual leaders showed a significant and positive linkage to job satisfaction (e.g., Haslam et al., 2021; Zacher and Rudolph, 2021). This showed a consensus regarding positive work outcomes by enhancing organizational psychology elements. Provision of support, autonomy, and resilience (through training and development programs and virtual social groups) are among the actions that leaders can undertake to establish trust to mediate job satisfaction as a positive psychological work outcome as demonstrated in the current results. Nurturing trust can lead to higher rates of job satisfaction as it fosters insecurity, stress, anxiety, and factors such as work engagement in virtual teams (e.g., Bradley et al., 2019; Haar and Brougham, 2020; Rashmi and Kataria, 2021; Telyani et al., 2021; Farmanesh and Zargar, 2023). These findings suggest the appropriate virtual leadership approach in the post-pandemic era calls for tech-savvy leaders.

This research emphasizes the role of leaders in creating trust in virtual teams while stressing the importance of WLB, especially for high-performance organizations (e.g., IT) in times of crises. Our results showed that the majority of the respondents were relatively young (see Table 1) and required more attention and care as anxiety, stress, and other negative factors from the COVID-19 pandemic affected their lives (professionally and personally) (Qiu and Dauth, 2021; Rashmi and Kataria, 2021; Telyani et al., 2021). In addition, employees of this section have faced an increasing amount of clients and projects, which has further diminished their WLB and job satisfaction. We found that through an environment of trust fostered by the leader, alongside promoting the wellbeing of employees by providing support and the means to create a balance between their work and life, job satisfaction can be increased as a positive work outcome with psychological impacts that go beyond the work environment.

Our results showed the vital role played by trust in the leader in improving job satisfaction. This was in line with previous studies from different contexts, which can be beneficial for scholars and practitioners (e.g., Braun et al., 2013; Castellano et al., 2021; Qiu and Dauth, 2021; Rashmi and Kataria, 2021; Farmanesh and Zargar, 2023). Linked to Maslow's hierarchy of needs, it is expected that virtual leaders address and meet the needs of their staff in times of crises. Virtual leaders accomplish such by fostering autonomy, competence, and support, which positively impact job satisfaction through WLB and trust as they provide motivation for employees, which can be further linked to the job demand–resource model (Poulose and Sudarsan, 2017; Dousin et al., 2019). Virtual teams pose a challenge for virtual leaders in terms of establishing trust due to the lack of direct interactions, distance, cultural differences, and a variety of communication channels. Hence, the role of leaders becomes more important in these settings and in uncertain times, like the health crisis presented by COVID-19 (Kudyba, 2020; Castellano et al., 2021), and perhaps in future crises.

### Practical implications

Leaders of IT organizations should be able to tap into different leadership styles to match the current status of their organizations and show competence in leading complex work environments. This can lead to innovative methods to conduct their leadership tasks, further benefiting organizational goals through the facilitation and development of positive work environments to obtain positive work outcomes in concert with improving employees' quality of life. Employees' personal and professional improvements can be achieved by leaders' initiatives to improve communication, provide support and mentorship, and design opportunities for progress at work. Collaboration and integration of leadership strategies with the HR departments should be among the main focuses of virtual leaders to positively establish psychological support. This allows the firm to prepare resilient programs and frameworks, provide support systems unique to each individual, dedicate incentives and wellbeing bonuses, and establish learning environments in which employees can improve both the professional and personal aspects of their lives, thus positively balancing work and life. As noted earlier, non-monetary bonuses and incentives can further influence job satisfaction, which the leaders can tailor based on the values and needs of each member.

The provision of autonomy and virtual social groups can also be among the initiatives that the E-leaders carry out to benefit their followers by lowering their anxiety, stress, and feelings of social exclusion. Emphasizing trust, virtual leaders can create a sense of identity among members who are physically distant from one another. Leaders can create a positive and shared set of values that incorporates policies of WLB and wellbeing and motivates performance and positive virtual work ethics. Leaders should focus on trust-building in virtual teams to improve the environment of work for all employees. In addition, the HR departments can create developmental tools (online courses, webinars, and other activities) to provide professional opportunities for staff. This can encourage growth and improve the job satisfaction of employees by paving future career opportunities. Leaders can engage in mentorship, counseling, and trust-building behavior with their staff in an individual manner to foster positive emotions through personalized care.

### Limitations and recommendations

As the data for this study were collected through a cross-sectional approach, future studies can deploy longitudinal data collection to account for variations in time as leaders introduce new elements in their virtual teams. Similarly, qualitative methods can provide a thorough understanding of the subject at hand by shedding light on themes and vital points from employee perspectives or leader perspectives. In addition, stress theories (e.g., the transactional stress model) can be integrated into future models to further examine the relevant factors and theoretical frameworks. Initiatives that specifically address trust can also be regarded in future studies due to the importance of this construct. The effect of the control variables of this research can be examined in different contexts. Furthermore, underlying mediator or moderator variables that can enhance virtual job satisfaction can be also examined to provide better solutions for virtual leaders.

## Data availability statement

The raw data supporting the conclusions of this article will be made available by the authors, without undue reservation.

## Ethics statement

Ethical review and approval was not required for the study on human participants in accordance with the local legislation and institutional requirements. Written informed consent from the patients/ participants or patients/participants legal guardian/next of kin was not required to participate in this study in accordance with the national legislation and the institutional requirements.

## Author contributions

PF: supervision, contextualization, and initial draft. HA: final draft, data collection, and analysis. All authors contributed to the article and approved the submitted version.
